# Thyroid dysfunctions and autoimmunity in breast cancer patients: a prospective case-control study

**DOI:** 10.20945/2359-3997000000284

**Published:** 2020-08-24

**Authors:** Chandan Kumar Jha, Anjali Mishra, Subhash B. Yadav, Gaurav Agarwal, Shalini Singh, Gyan Chand, Amit Agarwal, Saroj Kanta Mishra

**Affiliations:** 1 Sanjay Gandhi Postgraduate Institute of Medical Sciences Department of Endocrine Surgery Lucknow India Department of Endocrine Surgery, Sanjay Gandhi Postgraduate Institute of Medical Sciences, Lucknow, India; 2 All India Institute of Medical Sciences Department of General Surgery Patna India Department of General Surgery, All India Institute of Medical Sciences, Patna, India; 3 Sanjay Gandhi Postgraduate Institute of Medical Sciences Department of Medical Endocrinology Lucknow India Department of Medical Endocrinology, Sanjay Gandhi Postgraduate Institute of Medical Sciences, Lucknow, India; 4 Sanjay Gandhi Postgraduate Institute of Medical Sciences Department of Radiotherapy Lucknow India Department of Radiotherapy, Sanjay Gandhi Postgraduate Institute of Medical Sciences, Lucknow, India

**Keywords:** Hypothyroidism, Thyroid autoimmunity, Neoadjuvant chemotherapy, Breast cancer

## Abstract

**Objective:**

The relationship of thyroid dysfunction and autoimmunity with breast cancer (BC) continues to be contentious. The primary aim of this study was to estimate the prevalence of thyroid dysfunctions and autoimmunity in BC patients, and the secondary aims were to investigate the relationship of thyroid dysfunction with the clinicopathological profile of and therapy received by BC patients.

**Materials and methods:**

This was a single-center prospective case-control study (March 2015-May 2017). Women with BC (n = 191), age-matched healthy controls (n = 166) and malignant controls (patients with cervical cancer, n = 87) were enrolled. Basal serum free thyroxin (fT4), thyrotropin (TSH) and anti-thyroid peroxidase (TPO) antibody levels were measured in all three groups; fT4, TSH and TPO measures were repeated after chemotherapy and at the 1-year follow-up (one year after diagnosis) in the BC patients.

**Results:**

The prevalence of overall hypothyroidism and autoimmunity (p = 0.106) did not differ significantly between the three groups, but the rate of clinical hypothyroidism was significantly higher in the BC group than in the healthy control group and the malignant control group (12.2% *vs.* 3.0% *vs.* 4.6%, respectively; p = 0.001). BC patients had significantly lower mean basal TSH concentrations than the healthy controls (p = 0.017). The postchemotherapy TSH concentrations were significantly lower (p = 0.001), and the fT4 concentrations were higher, albeit not significantly (p = 1.00), than the respective basal concentrations. The reverse was true for the follow-up values, in which the TSH (p = 1.00) values were higher and the fT4 (p = 0.03) concentrations were lower than the respective basal concentrations. An additional 6% of the BC patients developed clinical hypothyroidism during follow-up. Hypothyroid (p = 0.02) and TPO-positive (p = 0.004) patients had significantly smaller tumors, but their other clinicopathological features were comparable to those without thyroid dysfunction.

**Conclusions:**

The prevalence of clinical hypothyroidism requiring thyroxine replacement was significantly high in BC patients and increased further during follow-up. Hence, BC patients should be considered a high-risk group that should receive routine screening for hypothyroidism.

## INTRODUCTION

Thyroid and breast tissue share some common molecular markers. Both normal and pathologic breast tissues exhibit high concentrations of sodium-iodide symporter, peroxidase, and deiodinase, indicating the active involvement of breast tissues in iodine metabolism ( 1 , 2 ). There is considerable conflicting literature on the relationship between thyroid and breast disorders ( 3 – 8 ). Two meta-analyses were published on this subject in 2012. While one of them suggested a positive correlation of breast cancer (BC) with thyroid autoimmunity and goiter, the other had contrary findings ( 9 , 10 ). The results of two recent meta-analyses were also equally conflicting. One suggested that thyroid dysfunction is not related to an increased risk of BC, whereas the other, which analyzed the effect of thyroid dysfunction on site-specific cancer risk, concluded that thyroid dysfunction was associated with an increased risk of BC ( 11 , 12 ). Whether hypothyroidism is a risk factor for the development of BC or a sequelae of multimodal cancer therapy is also not clear ( 7 , 13 – 15 ). The reported prevalence of thyroid dysfunction and autoimmunity in BC ranges from 9.3% to 56%. Some studies have indicated that BC patients with hypothyroidism have less aggressive tumors and better prognosis ( 7 , 16 – 18 ); on the contrary, the response to chemotherapy remains poor in these patients ( 13 , 19 ). The majority of the published studies on this subject are either cross-sectional or retrospective. Despite the significant short- and long-term clinical implications of hypothyroidism, there is no consensus regarding the routine screening of thyroid dysfunctions in patients with BC. We planned this prospective case-control study to clarify some of these issues in our patient population. The primary aim of this study was to estimate the prevalence of thyroid dysfunctions and autoimmunity in BC patients, and the secondary aims were to investigate the relationship of thyroid dysfunction with the clinicopathological profiles and therapies of the patients with BC.

## MATERIALS AND METHODS

This was a prospective longitudinal case-control study conducted at the authors’ institute (an academic institution). The Institute Ethics Committee approved the study (IEC code: 2014-125-IMP-79).

### Patients

The study included three groups of patients: a BC group and two control groups (an age-matched healthy control group [(HC] and a malignancy group [MC]). The BC group consisted of 191 consecutive newly diagnosed BC patients presenting at our institution between March 2015 and May 2017. Male BC patients, recurrent BC patients, pregnant patients and BC patients with liver disease were excluded. The HC group (n = 166) was recruited from among the relatives and friends of the BC patients who volunteered for participation, whereas the MC group (n = 87) consisted of patients with cervical cancer undergoing chemotherapy and/or radiotherapy.

### Thyroid function assessment

All BC patients underwent assessment of their thyroid function and autoimmunity three times- prior to initiation of treatment (basal sample), 3-4 weeks after completion of chemotherapy (postchemotherapy sample), and 3 months after completion of primary therapy including surgery, chemotherapy ± radiotherapy (follow-up sample). The HC and MC groups had assessments of thyroid function and autoimmunity completed only once. Serum TSH, serum fT4 and serum anti-TPO antibody estimations were performed on all blood samples using the IMMULITE^®^ and IMMULITE^®^1000 systems by Siemens with the prescribed kits. Centrifuged serum samples were stored at −20 °C for no more than one month. Preparation, setup, dilutions, adjustment assay and quality control procedures were performed as per the instructions in the operator's manual. The IMMULITE/IMMULITE 1000 third-generation TSH assay is a solid-phase, two-site, chemiluminescent immunometric assay, whereas the IMMULITE/IMMULITE 1000 Free T4 assay is a solid-phase, enzyme-labeled chemiluminescent competitive immunoassay, and the IMMULITE/IMMULITE 1000 Anti-TPO Ab assay is a solid-phase, enzyme-labeled chemiluminescent sequential immunometric assay.

### Definitions and outcome measures

- Clinical hypothyroidism: Elevated TSH levels beyond the reference range (0.4-4.0 μIU/mL) with subnormal fT4 levels (11.5-22.7 pmol/L).
- Subclinical hypothyroidism: Elevated TSH levels beyond the reference range with fT4 levels within the normal range.
- Hyperthyroidism: Patients with TSH levels < 0.01 μIU/mL were considered to have hyperthyroidism, those with normal fT4 levels were considered to have subclinical hypothyroidism, and those with fT4 above the reference range were considered to have clinical hyperthyroidism.
- Autoimmunity: Serum anti-TPO antibody level > 35 IU/L.
- The primary outcome measure was hypothyroidism (subclinical + clinical).

### Follow-up protocol

Subjects (BC cases, HC, and MC) with clinical hypothyroidism and those with subclinical hypothyroidism with TSH levels > 10 μIU/mL were put on a thyroxine supplement. TSH and T4 measurements were repeated at 6-week intervals and thyroxine dosages were adjusted accordingly. Once the values were within normal limits, a repeat measurement was performed prior to surgery and annually thereafter. Other subjects with subclinical hypothyroidism and TSH levels < 10 μIU/mL and normal fT4 levels were evaluated prior to surgery and annually thereafter; they were followed in a specialized endocrine clinic per standard guidelines ( 20 ). Two patients who had thyrotoxicosis, one in the BC cohort and another in the HC cohort, were found to have subacute thyroiditis and were managed accordingly. Controls (HC and MC) that did not have thyroid dysfunction/autoimmunity were not followed any further.

### Statistical analysis

The number of study subjects was calculated based on our previous study on benign breast disease (BBD) patients, in which the prevalence of hypothyroidism was found to be 23.3% ( 21 ). Based on this survey and published reports, the maximum prevalence of hypothyroidism in the general population was estimated to be 20% ( 22 ). With an anticipated prevalence of 35% in BC patients and the study power set at 90% with a significance level of 0.05, a minimum sample size of 185 was calculated.

The proportions and mean difference in laboratory values were compared between the case and control groups. In the BC group, the differences among basal, postchemotherapy and follow-up values were compared. Thyroid dysfunction and autoimmunity were correlated with various clinicopathological factors: age, menopausal status, tumor size, grade, lymph nodal status, distant metastases, hormone receptor status, and HER-2 status. Chi-square tests, Student's t-tests, Mann-Whitney tests, and log-rank tests were used as indicated. The p-values were adjusted (Bonferroni method) for multiple comparisons.

### Management of breast cancer

BC was staged according to AJCC staging and managed according to standard guidelines and departmental protocols. Chemotherapy response was judged by the RECIST criteria ( 23 ).

## RESULTS

None of the 191 BC patients included in the study had screen-detected tumors and all presented with a lump in the breast. Seventy-three percent of patients presented with axillary lymph node involvement, and six percent of patients presented with distant metastases. Baseline samples collected from all 191 patients, postchemotherapy samples collected from186 patients, and follow-up samples collected from142 patients were available for analysis. All MC were receiving radiotherapy and/or chemotherapy at the time of sample collection.

### Prevalence of thyroid dysfunction and autoimmunity

In this study, all patients with thyroid dysfunction had hypothyroidism (clinical or subclinical), except one patient each in the BC group and HC group who had thyrotoxicosis. The prevalence of thyroid dysfunction was similar in all three groups (29.8%, 29.4%, and 25.2% in the BC, HC, and MC groups, respectively; p = 0.90). The prevalence of autoimmunity was also similar in all three groups (14.1%, 22.9%, and 14.9%in the BC, HC, and MC groups, respectively; p = 0.106). A total of 6.8% of patients in the BC group had evidence of both hypothyroidism and autoimmunity, and 37.2% of patients had either/or both abnormalities ( Table 1 ). The prevalence of clinical hypothyroidism was significantly higher in the BC group than in the HC or MC groups (12.2% *vs.* 3.0% *vs.* 4.6%, respectively; p = 0.001).

**Table 1:** Thyroid dysfunction and autoimmunity in the three groups

Sl No			Breast Cancer Cases (n = 191)	Healthy Controls (n = 166)	Malignant Controls (n = 87)	p-value
1.	Age in years: Mean ± SD (range)		47.6 ± 10.4 (26-78)	45.8 ± 9.5 (30-75)	53.1 ± 7.7 (34-75)	0.094 *
2.	Hypothyroidism: n (%)		56 (29.3)	46 (28.8)	22 (25.2)	0.900
		Clinical Hypothyroidism: n (%)	24 (12.6)	05 (3.0)	04 (4.6)	0.001 **
		Hyperthyroidism: n (%)	01 (0.5)	01 (0.6)	−	
5.	Autoimmunity: n (%)		27 (14.1)	36 (22.9)	16 (14.9)	0.106
3.	fT4 (pmol/L): Mean ± SD (range)		16.1 ± 3.7 (4.4 -34.0)	15.5 ± 2.9 (10-33.2)	15.1± 2.7 (8.9-23.9)	0.050 ***
4.	TSH (μIU/mL): Mean ± SD (range)		2.7 ± 2.2 (0.02- 16.0)	3.4 ± 2.5 (0.01-14.0)	3.0 ± 3.0 (0.12-16.7)	0.035 ****

* Breast cancer cases and Healthy Controls.

** Significant difference only in self-reported and clinical hypothyroidism prevalence.

*** Breast cancer cases *vs.* Healthy controls, p = 0.204; Breast cancer cases *vs.* Cervical cancer cases, p = 0.047; Healthy controls *vs.* Cervical cancer cases, p = 1.000.

**** Breast cancer cases *vs.* Healthy controls, p = 0.017; Breast cancer cases *vs.* Cervical cancer cases, p = 1.000; Healthy controls *vs.* Cervical cancer cases, p = 0.510.

### Effect of breast cancer therapy on thyroid function and autoimmunity

All BC patients included in this study received chemotherapy. Sixty-two percent of the patients received neoadjuvant chemotherapy (anthracyclines followed by taxanes), and the remaining patients received adjuvant chemotherapy. Ninety percent of the patients also received adjuvant radiotherapy.

A summary of the mean basal values of fT4 and TSH in the BC group is provided in Table 1 . The postchemotherapy mean fT4 concentration was higher, though not significantly (p = 1.000), and the mean TSH level was significantly lower (p= 0.001) than the basal concentration, whereas the mean follow-up fT4 concentration was significantly lower (p = 0.030) and TSH was higher, though not significantly (p = 1.000) than the basal value. Similar observations were noted when analyzing the euthyroid and hypothyroid groups of BC patients separately ( Table 2 ). An additional six percent (n = 13) of the BC patients developed clinical hypothyroidism at the end of one year, thus increasing the prevalence of clinical hypothyroidism in the BC group to 18.3%. The autoimmunity status of the BC patients did not change after chemotherapy or even after completion of treatment (1 year from the time of diagnosis). No further follow-up information was recorded for any of the patients in this study.

**Table 2:** Serum fT4 and TSH values in various subgroups of BC patients

Sl No	Group/Subgroup	Measure	Basal	Postchemotherapy	Follow-up	p-value
1.	All patients (191/186/142 # )	- fT4 (pmol/L)	15.8 ± 2.8	16.1 ± 3.5	15.1 ± 3.2	0.054
		- TSH (µIU/mL)	2.7 ± 2.4	2.0 ± 1.6	3.1 ± 5.5	0.004
2.	Euthyroid patients (134/131/101)	- fT4 (pmol/L)	16.2 ± 2.8	16.0 ± 3.1	15.2 ± 3.4	0.038
		- TSH (µIU/mL)	1.7 ± 1	1.6 ± 1.3	2.8 ± 5.6	0.019
3.	Hypothyroid patients (56/55/41)	- fT4 (pmol/L)	15.1 ± 3.5	16.2 ± 4.0	14.8 ± 2.6	0.509
		- TSH (µIU/mL)	5.1 ± 2.7	2.7 ± 1.7	3.9 ± 5.3	0.010
4.	Sub-Clinical Hypothyroid patients (32/24/22)	- fT4 (pmol/L)	14.3 ± 1.8	16.0 ± 4.2	14.5 ± 3.1	0.902
		- TSH (µIU/mL)	5.7 ± 1.7	2.9 ± 1.9	4.8 ± 7.1	0.003

# Number of patients: basal/postchemotherapy/follow-up.

Basal *vs.* Postchemotherapy, Postchemotherapy *vs.* Follow-up, and Basal *vs.* Follow-up p-values, respectively, for fT4 and TSH in rows 1-4.
fT4- 1.000, 0.021, 0.030; TSH-0.001, 0.051, 1.000. fT4- 0.453, 0.075, 0.009: TSH- 0.192, 0.009, 0.231. fT4- 0.207, 0.366, 1.000; TSH-0.001, 1.000, 0.024. fT4-0.819, 0.651, 1.000; TSH- 0.001, 0.897, 1.000.

### Thyroid dysfunction and clinicopathological profile of the BC patients

The clinicopathological features of the BC patients with and without thyroid dysfunction and autoimmunity are summarized in Tables 3 and 4 , respectively. Hypothyroid (5.3 *vs.* 4.5 cm, p = 0.013) and TPO positive (5.2 *vs.* 4.0 cm, p = 0.006) patients had significantly smaller tumors than their counterparts, though there was no difference in the mean duration of symptoms. TPO-positive patients were less likely to have T4 tumors (p = 0.038) and more likely to undergo breast-conserving surgery (p = 0.008). Other clinicopathological prognostic and predictive factors were not significantly different in patients with or without thyroid dysfunction or autoimmunity.

**Table 3:** Clinical and pathologic features of breast cancer in patients with euthyroidism and thyroid dysfunction

Sl No	Feature	Euthyroid Dysfunction Patients (n = 134)	Hypothyroid Dysfunction Patients (n = 56)	p-value
1.	Age in years: Mean ± SD	46.9 ± 10.7	49.6 ± 9.3	0.102
2.	Duration in months: Mean ± SD	8.8 ± 14.6	5.6 ± 6.9	0.118
3.	Autoimmunity: n (%)	14 (10.4)	12 (21.4)	0.007 *
4.	Menopausal status: n (%) Pre/Post	58/76 (43.3/56.7)	19/37 (33.9/66.1)	0.236
5.	Tumor size in cm: Mean ± SD	5.3 ± 1.9	4.5 ± 2.1	0.013 *
6.	Tumor (%)	3/4/48/38/41	1/5/23/12/15	0.662
	Tx/T1/ T2/ T3/T4	(2.2/3.0/35.8/28.4/30.6)	(1.8/8.9/41.1/21.4/26.8)	
7.	Lymphadenopathy: n (%)	96 (71.6)	43 (76.8)	0.461
8.	Distant metastases: n (%)	7 (5.2)	5 (8.9)	0.610
9.	Tumor grade: n (%)	7/51/68/8	1/23/27/5	0.530
	1/2/3/Others	(5.2/38.1/50.7/5.9)	(1.8/41.1/48.2/8.9)	
10.	Lymphovascular invasion: n (%)	42 (31.6)	13 (23.6)	0.761
11.	Perineural invasion: n (%)	5 (3.8)	1 (1.8)	0.934
12.	ER positive: n (%)	64 (47.8)	18 (32.1)	0.267
	PR positive: n (%)	59 (44.0)	18 (32.1)	0.366
	HER2/NEU positive: n (%)	68 (50.7)	30 (53.6)	0.677
13.	Type of chemotherapy: n (%)	87/41/6	25/25/6	0.087
	NACT/Adjuvant/Palliative	(64.9/30.6/4.5)	(44.6/44/6/10.7)	
14.	Responders to chemotherapy a : n (%)	78 (87.6)	22 (88)	0.931
15.	Surgery: n (%)	127 (94.8)	51 (91.1)	0.610
	MRM/BCS b	104/24 (81.2/18.8)	40/11 (78.4/21.6)	0.807
16.	Radiotherapy: n (%)	123 (91.8)	47 (83.9)	0.256

* Significant values.

a Applicable to 115 patients who received neoadjuvant chemotherapy.

b MRM: modified radical mastectomy; BCS: breast conserving surgery.

**Table 4:** Clinical and pathologic features of breast cancer in patients with normal and raised TPO values

Sl No	Feature	Normal TPO (n = 164)	Raised TPO (n = 27)	p-value
1.	Age in years: Mean ± SD	47.8 ± 10.5	46.0 ± 9.7	0.394
2.	Duration in months: Mean ± SD	8.3 ± 13.7	5.0 ± 5.0	0.215
3.	Hypothyroidism: n (%)	44 (26.8)	12 (44.4)	0.007 *
4.	Menopausal status: n (%) (pre/post)	69/95 (42.1/57.9)	11/16 (30.7/59.3)	0.392
5.	Tumor size in cm: Mean ± SD	5.2 ± 2.0	4.0 ± 1.4	0.006 *
6.	Tumor (%)	4/7/56/43/54	0/2/16/7/2	0.038 *
	Tx/T1/T2/T3/T4	(2.4/4.3/34.1/26.2/32.9)	(0/7.4/59.3/25.9/7.4)	
7.	Lymphadenopathy (%)	119 (72.6)	20 (74.1%)	0.914
8.	Distant Metastases: n (%)	12 (7.3)	0	0.147
9.	Tumor Grade: n (%)	7/67/78/12	1/7/17/2	0.483
	½/3/Others	(4.3/40.9/47.6/7.3)	(3.7/25.9/63/7.4)	
10.	Lymphovascular invasion: n (%)	45 (27.6)	10 (38.5)	0.257
11.	Perineural invasion: n (%)	05 (3.1)	01 (3.8)	0.390
12.	ER positive: n (%)	69 (42.1)	13 (48.1)	0.663
	PR positive: n (%)	65 (39.6)	13 (48.1)	0.543
	HER2/NEU positive: n (%)	85 (51.8)	13 (48.1)	0.412
13.	Type of chemotherapy: n (%)	98/54/12	15/12/0	0.231
	NACT/Adjuvant/Palliative	(59.8/32.9/7.3)	(55.6/44.4/0)	
14.	Responders to chemotherapy a : n (%)	90 (88.2)	11 (84.6)	0.658
15.	Surgery: n (%)	153 (93.3)	26 (96.3)	0.551
	MRM/BCS b	129/24 (83.8/16.2)	17/9 (61.5/38.5)	0.008 *
16.	Radiotherapy: n (%)	145 (88.4)	26 (96.3)	0.215

* Significant values.

a Applicable to 115 patients who received neoadjuvant chemotherapy.

b MRM: modified radical mastectomy; BCS: breast conserving surgery.

## DISCUSSION

The current study shows that in comparison to controls (age-matched normal and with malignancy), the prevalence of overall hypothyroidism (subclinical and clinical) and autoimmunity was not higher in BC patients, although the prevalence of clinical hypothyroidism remained significantly high. BC patients with thyroid dysfunctions and autoimmunity had significantly smaller tumors than those without dysfunction and autoimmunity. In comparison to basal values, mean fT4 was higher and TSH was lower after chemotherapy but after one year of follow-up, the reverse trend was noted, i.e., lower fT4 and higher TSH than the respective values at baseline.

The findings of a significantly high prevalence of clinical hypothyroidism and significantly smaller tumors in BC patients seem related. This can partly be explained by the fact that the patients with self-reported hypothyroidism were already under medical care and were perhaps more aware to seek early consultation for breast lumps, which are otherwise often ignored for a long time in our country. The higher Prevalence of self-reported hypothyroidism in our BC cohort is similar to the rates reported in previous studies ( 3 , 5 ). Approximately 9.9% of patients in the current study were taking a thyroxine replacement, which seems on par with the 9.3% reported by Adamopoulos and cols. ( 3 ). The overall prevalence of hypothyroidism was higher in BC patients than benign breast disease patients, as was reported in our previous study ( 21 ). However, contrary to published reports, the prevalence of autoimmunity was not found to be high in BC patients. Previous studies have reported higher rates of thyroid microsomal, TPO and thyroglobulin antibody (TgAb) antibodies in BC patients ( 2 , 3 , 5 , 8 , 9 ).

The finding of a lower mean basal TSH concentration in BC patients than in the control groups is in contrast to previously published studies ( 3 , 5 ). We do not know the exact explanation for this finding, but it could be multi-factorial; low TSH level alone has been considered a risk factor for the development of various malignancies, and a recent meta-analysis showed that hyperthyroidism is associated with a higher risk of breast and other cancers ( 12 , 13 ). Many previous studies also did not include age-matched controls, making this comparison difficult ( 3 ). Postchemotherapy alterations in fT4 and TSH values could be the result of delayed thyroid hormone clearance caused by an effect of chemotherapy, as also observed by other researchers ( 19 , 24 ).

The reason for the high prevalence of clinical hypothyroidism in BC patients seems to also be multifactorial. It is postulated that malignancy per se could be a predisposing condition for hypothyroidism ( 13 – 15 , 25 – 27 ). The occurrence of hypothyroidism in cancer is attributed to various cytokines secreted by the tumor that inhibit the function of the hypothalamus-pituitary-thyroid axis and peripheral conversion of T 4 to T3. The high prevalence of hypothyroidism in BC survivors has also been attributed to the multimodality therapy employed in treating the malignancy ( 6 , 28 ). Both chemotherapy and radiotherapy have the propensity to induce hypothyroidism ( 13 , 19 , 28 – 30 ). The median time for the development of hypothyroidism after radiotherapy ranges from 3 to 4.5 months. A study based on the surveillance, epidemiology, and end results database found that irrespective of radiotherapy status, the prevalence of hypothyroidism was higher in BC survivors. The 1- and 5-year risk of developing hypothyroidism was 4% and 14%, respectively, after BC diagnosis, whereas the corresponding figures for the controls were 2% and 11%, respectively ( 29 ). A recent meta-analysis also reported that the thyroid gland is affected by radiotherapy, resulting in a significant increase in serum TSH ( 31 ). In the current study, both the BC and MC groups had comparable prevalence rates of hypothyroidism. MC patients were already undergoing therapy. Their mean fT4 values were lower and their TSH values were higher than those of the HC. The BC patients also showed a similar trend in follow-up, during which an additional 9.9% of the BC patients developed hypothyroidism, hinting at the role of multimodality therapy.

Some studies have also shown that BC patients with hypothyroidism have smaller tumors, less aggressive disease and a better prognosis ( 7 , 13 , 16 – 18 , 31 ). Women with TPO and antithyroid antibodies are reported to have a better prognosis than those lacking these antibodies ( 16 – 18 ). In the current study, BC patients with hypothyroidism and autoimmunity had significantly smaller tumors and were likely to have a better prognosis, but we cannot conclusively comment on the long-term prognosis. As previously mentioned, this could be related to more intense follow-up in the patients with hypothyroidism. It has been reported that the response to chemotherapy is poor in patients with hypothyroidism ( 13 , 19 ). However, we did not find any difference in chemotherapy response between the euthyroid and hypothyroid patients.

Both hypothyroidism and BC are highly prevalent among females; also, the occurrence of both diseases increases with age, making it difficult to establish a real correlation between them. Additionally, the BC prevalence might have been overestimated due to a higher rate of medical consults and visits in the group of thyroid patients than in the general population. The majority of the published studies to date was cross-sectional and lacked a properly matched control population. Therefore, to definitively investigate this debate, a larger and longer well designed and properly conducted prospective study is critical.

While a cause-effective relationship between BC and hypothyroidism has yet to be demonstrated hypothyroidism does seem to be a significant clinical problem in association in BC, and yet, there is no consensus for routine screening of thyroid dysfunctions in BC patients. We suggest that routine screening of thyroid function should be included in the evaluation of and follow-up protocol for BC patients. This would enable us to identify the patients who need thyroxine replacement and thus avoid the perioperative and long-term complications of hypothyroidism.

The strength of this study is that, unlike previous studies, the same cohort of newly diagnosed BC patients was followed to study the basal, postchemotherapy, and follow-up thyroid hormone status. The major weakness is that the follow-up was short, i.e., the duration of follow-up was limited to one year from the diagnosis of BC. A longer follow-up could result in a further increased incidence of overall and/or clinical hypothyroidism.

In conclusion, the prevalence of clinical hypothyroidism is significantly high in BC patients and increases further in follow-up. We suggest that BC patients should be considered a high-risk group that should receive routine screening for hypothyroidism.

## References

[B1] Venturi S. Is there a role for iodine in breast diseases? Breast. 2001;10:379-82. 10.1054/brst.2000.0267 14965610

[B2] Muller I, Barrett-Lee PJ. The antigenic link between thyroid autoimmunity and breast cancer. Semin Cancer Biol. 2019. pii: S1044-579X(19)30043-4. 10.1016/j.semcancer.2019.05.013 31128301

[B3] Adamopoulos DA, Vassilaros S, Kapolla N, Papadiamantis J, Georgiakodis F, Michalakis A. Thyroid disease in patients with benign and malignant mastopathy. Cancer. 1986;57:125-8. 10.1002/1097-0142(19860101)57:1<125::aid-cncr2820570125>3.0.co;2-4 3940612

[B4] Shering SG, Zbar AP, Moriarty M, McDermott EW, O’Higgins NJ, Smyth PP. Thyroid disorders and breast cancer. Eur J Cancer Prev. 1996;5:504-6. 9061284

[B5] Giustarini E, Pinchera A, Fierabracci P, Roncella M, Fustaino L, Mammoli C, et al. Thyroid autoimmunity in patients with malignant and benign breast diseases before surgery. Eur J Endocrinol. 2006;154:645-9. 10.1530/eje.1.02108 16645010

[B6] Saraiva PP, Figueiredo NB, Padovani CR, Brentani MM, Nogueira CR. Profile of thyroid hormones in breast cancer patients. Braz J Med Biol Res. 2005;38:761-5. 10.1590/s0100-879x2005000500014 15917958

[B7] Cristofanilli M, Yamamura Y, Kau SW, Bevers T, Strom S, Patangan M, et al. Thyroid hormone and breast carcinoma. Primary hypothyroidism is associated with a reduced incidence of primary breast carcinoma. Cancer. 2005;103:1122-8. 10.1002/cncr.20881 15712375

[B8] Sarlis N, Gourgiotis L, Pucino F, Tolis GT. Lack of association between Hashimoto thyroiditis and breast cancer: a quantitative research synthesis. Hormones (Athens). 2002;1:35-41. 10.14310/horm.2002.1152 17018436

[B9] Hardefeldt PJ, Eslick GD, Edirimanne S. Benign thyroid disease is associated with breast cancer: a meta-analysis. Breast Cancer Res Treat. 2012;133:1169-77. 10.1007/s10549-012-2019-3 22434524

[B10] Angelousi AG, Anagnostou VK, Stamatakos MK, Georgiopoulos GA, Kontzoglou KC. Mechanisms in Endocrinology: Primary HT and risk for breast cancer: a systematic review and meta-analysis. Eur J Endocrinol. 2012;166:373-81. 10.1530/EJE-11-0838 22023791

[B11] Fang Y, Yao L, Sun J, Yang R, Chen Y, Tian J, et al. Does thyroid dysfunction increase the risk of breast cancer? A systematic review and meta-analysis. J Endocrinol Invest. 2017;40:1035-47. 10.1007/s40618-017-0679-x 28516372

[B12] Tran TV, Kitahara CM, de Vathaire F, Boutron-Ruault MC, Journy N. Thyroid dysfunction and cancer incidence: a systematic review and meta-analysis. Endocr Relat Cancer. 2020;ERC-19-0417.R2. 10.1530/ERC-19-0417 32045361

[B13] Moeller LC, Führer D. Thyroid hormone, thyroid hormone receptors, and cancer: a clinical perspective. Endocr Relat Cancer. 2013;20:R19-29. 10.1530/ERC-12-0219 23319493

[B14] Reinertsen KV, Cvancarova M, Wist E, Bjøro T, Dahl AA, Danielsen T, et al. Thyroid function in women after multimodal treatment for breast cancer stage II/III: comparison with controls from a population sample. Int J Radiat Oncol Biol Phys. 2009;75:764-70. 10.1016/j.ijrobp.2008.11.037 19286332

[B15] Koc M, Capoglu I, Unuvar N. Does the tamoxifen increase thyroid dysfunction after loco-regional irradiation of breast cancer? Radiother Oncol. 2001;59:361-2. 10.1016/s0167-8140(01)00284-5 11432373

[B16] Muller I, Giani C, Zhang L, Grennan-Jones FA, Fiore E, Belardi V, et al. Does thyroid peroxidase provide an antigenic link between thyroid autoimmunity and breast cancer? Int J Cancer. 2014;134:1706-14. 10.1002/ijc.28493 24114667

[B17] Fiore E, Giustarini E, Mammoli C, Fragomeni F, Campani D, Muller I, et al. Favorable predictive value of thyroid autoimmunity in high aggressive breast cancer. J Endocrinol Invest. 2007;30:734-8. 10.1007/BF03350810 17993764

[B18] Sandhu MK, Brezden-Masley C, Lipscombe LL, Zagorski B, Booth GL. Autoimmune hypothyroidism and breast cancer in the elderly. Breast Cancer Res Treat. 2009;115:635-41. 10.1007/s10549-008-0104-4 18604583

[B19] Huang J, Jin L, Ji G, Xing L, Xu C, Xiong X, et al. Implication from thyroid function decreasing during chemotherapy in breast cancer patients: chemosensitization role of triiodothyronine. BMC Cancer. 2013;13:334. 10.1186/1471-2407-13-334 PMC3717040 23829347

[B20] Garber JR, Cobin RH, Gharib H, et al. Clinical practice guidelines for hypothyroidism in adults: Cosponsored by the American Association of Clinical Endocrinologists and the American Thyroid Association. Endocrine PracT. 2012;18:988-1028. 10.4158/EP12280.GL 23246686

[B21] Bhargav PR, Mishra A, Agarwal G, Agarwal A, Verma AK, Mishra SK. Prevalence of Hypothyroidism in Benign Breast Disorders and Effect of Thyroxine Replacement on the Clinical Outcome. World J Surg. 2009;33:2087-93. 10.1007/s00268-009-0143-y 19641955

[B22] Yadav S, Gupta SK, Godbole MM, Jain M, Singh U, Pavithran PV, et al. Persistence of severe iodine-deficiency disorders despite universal salt iodization in an iodine-deficient area in northern India. Public Health Nutr. 2010;13:424-9. 10.1017/S1368980009990280 19519973

[B23] Eisenhauer EA, Therasse P, Bogaerts J, Schwartz LH, Sargent D, Ford R, et al. New response evaluation criteria in solid tumours: Revised RECIST guideline (version 1.1). Eur J Cancer. 2009;45:228-47. 10.1016/j.ejca.2008.10.026 19097774

[B24] Willemse PH, Sleijfer DT, Sluiter WJ, Koops HS, Doorenbos H. Alterations in thyroid hormone metabolism during chemotherapy in patients with testicular carcinoma. Clin Endocrinol (Oxf). 1982;16:303-13. 10.1111/j.1365-2265.1982.tb00720.x 6804138

[B25] López-Fontana CM, Sasso CV, Maselli ME, Santiano FE, Semino SN, Carrión FDC, et al. Experimental hypothyroidism increases apoptosis in dimethylbenzanthracene-induced mammary tumors. Oncol Rep. 2013;30:1651-60. 10.3892/or.2013.2648 23912381

[B26] Poth M, Tseng YC, Wartofsky L. Inhibition of TSH activation of human cultured thyroid cells by tumor necrosis factor: an explanation for decreased thyroid function in systemic illness? Thyroid. 1991;1:235-40. 10.1089/thy.1991.1.235 1668616

[B27] Pang XP, Yoshimura M, Hershman JM. Suppression of rat thyrotroph and thyroid cell function by tumor necrosis factor-alpha. Thyroid. 1993;3:325-30. 10.1089/thy.1993.3.325 8118227

[B28] Cutuli B, Quentin P, Rodier JF, Barakat P, Grob JC. Severe hypothyroidism after chemotherapy and locoregional irradiation for breast cancer. Radiother Oncol. 2000;57:103-5. 10.1016/s0167-8140(00)00183-3 11203360

[B29] Laway BA, Shafi KM, Majid S, Lone MM, Afroz F, Khan S, et al. Incidence of primary hypothyroidism in patients exposed to therapeutic external beam radiation, where radiation portals include a part or whole of the thyroid gland. Indian J Endocrinol Metab. 2012;16(Suppl 2):S329-31. 10.4103/2230-8210.104078 PMC3603064 23565416

[B30] Smith GL, Smith BD, Giordano SH, Shih YC, Woodward WA, Strom EA, et al. Risk of Hypothyroidism in Older Breast Cancer Patients Treated With Radiation. Cancer. 2008;112:1371-9. 10.1002/cncr.23307 18213620

[B31] Darvish L, Ghorbani M, Teshnizi SH, Roozbeh N, Seif F, Reza Bayatiani M, et al. Evaluation of thyroid gland as an organ at risk after breast cancer radiotherapy: a systematic review and meta-analysis. Clin Transl Oncol. 2018;20:1430-8. 10.1007/s12094-018-1875-7 29761266

